# Case report: Multiple brain intravascular papillary endothelial hyperplasia: incidence, diagnostic challenges, and management approach

**DOI:** 10.3389/fneur.2023.1115325

**Published:** 2023-04-20

**Authors:** Elena Anghileri, Bianca Pollo, Paolo Ferroli, Domenico Aquino, Greta Demichelis, Marco Schiariti, Francesco Ferrau, Luisa Chiapparini, Valeria Cuccarini

**Affiliations:** ^1^Neuroncology Unit, Fondazione Irccs Istituto Neurologico Carlo Besta, Milan, Italy; ^2^Neuropathological Unit, Fondazione Irccs Istituto Neurologico Carlo Besta, Milan, Italy; ^3^Neurosurgical Department, Fondazione Irccs Istituto Neurologico Carlo Besta, Milan, Italy; ^4^Neuroradiological Unit, Fondazione Irccs Istituto Neurologico Carlo Besta, Milan, Italy; ^5^Medical Oncology, Ospedale “S.Vincenzo”, Taormina, Italy; ^6^Radiodiagnostic Department, Fondazione IRCCS Policlinico San Matteo, Pavia, Italy

**Keywords:** intravascular papillary endothelial hyperplasia (IPEH), Masson's tumor, interferon, cerebral, sirolimus

## Abstract

Multiple hemorrhagic brain lesions are mainly diagnosed based on clinico-radiological features integrated with histological data. Intravascular papillary endothelial hyperplasia (IPEH), or Masson's tumor, is a very rare entity, particularly when localized in the brain. In this study, we describe a case of multiple recurrent brain IPEHs and provide details on the diagnostic phase, therapeutic approaches, and related challenges. A 55-year-old woman presented with a relapsing neurological deficit. Brain magnetic resonance imaging (MRI) revealed a hemorrhagic right frontal-parietal lesion. When new neurological symptoms occurred, subsequent MRI scans detected more bleeding cerebral lesions. She underwent a series of single hemorrhagic lesion debulking. For any samples that underwent histopathological examination, the first results were not informative; the second and the third results revealed hemangioendothelioma (HE); and the fourth results led to the IPEH diagnosis. Interferon alpha (IFN-α) and subsequently sirolimus were prescribed. Both were well tolerated. Clinical and radiological features remained stable 43 months after starting sirolimus therapy and 132 months after the first diagnosis. To date, 45 cases of intracranial IPEH have been reported, mostly as single lesions without parenchymal location. They are usually treated by surgery and sometimes by radiotherapy upon recurrence. Our case is notable for two main reasons: because of the consecutive recurrent multifocal exclusively cerebral lesions and the therapeutic approach we used. Based on multifocal brain recurrence and good performance, we propose pharmacological therapy, including IFN-α and sirolimus, to stabilize IPEH.

## Introduction

Multiple hemorrhagic brain lesions are primarily due to hypertension and cerebral amyloid angiopathy. Secondary causes include vascular malformations, coagulopathy, hemorrhagic conversion of ischemic stroke, hemorrhagic neoplastic localizations (metastases or vascular tumors), trauma, vasculitis, stimulant drugs, or sinus venous thrombosis (1). Vascular tumors of the brain are rare, and their main types are summarized in Table 1.

**Table 1:** The most important vascular tumors of the brain/head and neck.

**Benign**	**Locally aggressive/borderline**	**Malignant**
Infantile hemangioma Epithelioid hemangioma Papillary hemangioma	Hemangioblastoma (both sporadic and in von Hippel Lindau) Papillary intralymphatic angioendothelioma (Dabska tumor) Kaposiform hemangioendothelioma Tufted angioma Intravascular papillary endothelial hyperplasia (Masson's tumor) Hemangioendothelioma not otherwise specified	Angiosarcoma Hemangioendothelioma

Based on WHO Classification of Tumors Editorial Board. Soft tissue and bone tumors. Ed. 2020 (2).

Clinico-radiological features and histological reports of such hemorrhagic brain lesions are essential to make the diagnosis, but histologically, a definitive diagnosis has proven difficult because the hemorrhages destroy the tissue. Moreover, some vascular tumors may have similar or overlapping morphological features and immunophenotypes.

We describe a case of intravascular papillary endothelial hyperplasia (IPEH), or Masson's tumor (per the last revised *International Society for the Study of Vascular Anomalies (ISSVA) Classification*, https://www.issva.org/classification), a rare condition mostly localized in the skin and subcutaneous tissues. Our report is remarkable for noting an exclusively intracranial recurrent multifocal IPEH, stabilized during immunomodulation with interferon-alpha (IFN-α) and mechanistic target of rapamycin (mTOR) inhibitor treatment.

## Case report

**Figure 1:**
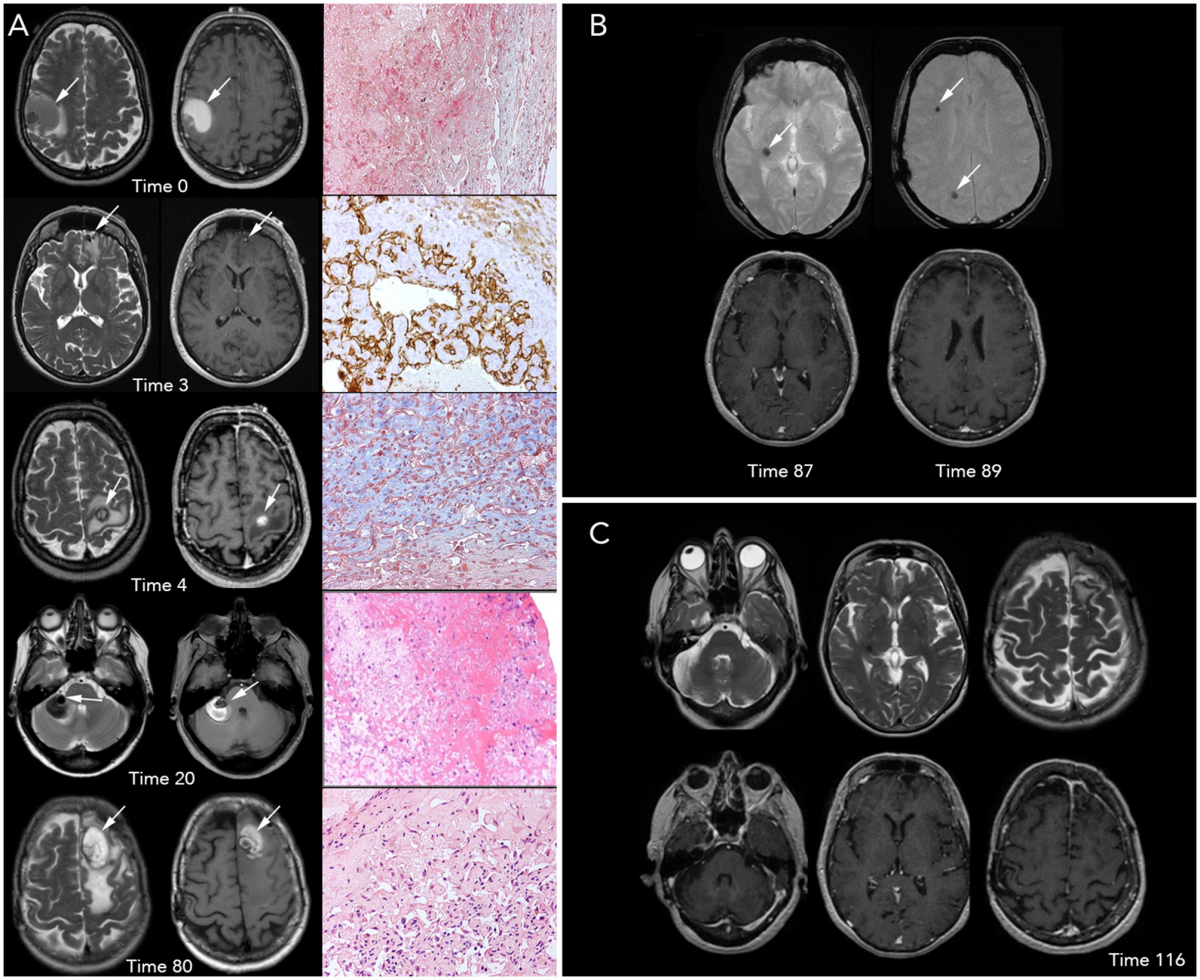
Brain magnetic resonance imaging (MRI) and histopathological imaging. **(A)** Left-to-right, each row includes T2- and T1-weighted images (w.i.) and histological features at precise time points. First row, Time 0 (onset): a large intra-axial hemorrhagic (T1 spontaneous hyperintensity) lesion surrounded by edema (T2 hyperintensity) is visible in the right frontal-parietal region. View of Masson Trichrome stain (original magnification x20) showing partially organized hematoma surrounded by granulation tissue. Second row, Time 3 months: three small, new intracranial hemorrhagic lesions with extensive edema are noted. The left frontal lesion, shown in the figure, was removed and underwent a histopathological examination, which is concluded as a hemangioendothelioma (HE); in particular, the tissue morphology was characterized by papillary structures covered by a single endothelial cell layer, expressing the endothelial-specific markers, as CD31 (x20). Third row, Time 4: a new left frontal hemorrhagic mass is documented; histology with Masson Trichrome (x20) revealed the fibrous wall of hematoma and an area consisting of vascular proliferation with papillary architecture compatible with HE. Fourth row, Time 20: a new right hemispheric cerebellar hemorrhagic lesion associated with mass effect is found. Low-magnification (x10) view of the hematoxylin and eosin stain of the lesion shows only a partially organized hematoma. Fifth row, Time 80: the figure shows a new large hemorrhagic mass in the left parasagittal frontal region surrounded by edema. Hematoxylin and eosin stain (x20) demonstrate a compact network of papillary structures with a collagenous axis, suggestive of intravascular papillary endothelial hyperplasia (IPEH, Masson's tumor). **(B)** Times 87 (left) and 89 (right): T2* w.i. (top row) show that three further lesions with hemosiderin deposition (T2* hypointensities) without recent-bleed hyperintensity in T1 w.i. (bottom row) in right internal subcapsular (Time 87) and frontal and parietal white matter (Time 89), respectively. Note: arrows show the new lesions on MRI images. **(C)** T2 w.i. (top) and contrast-enhanced T1 w.i. (bottom) at Time 116, after 27 months of treatment with sirolimus, showing no new lesions.

A 55-year-old Caucasian woman presented with the sudden onset of left facial-brachial syndrome (Time 0). In particular, she had dysesthesia of the left hand that, within 2 days, extended to the whole arm and half-face, which was associated with dysarthria. The latter symptom disappeared within a few days. She did not complain of having a headache, and the physiological parameters, including body temperature, blood arterial pressure, and heart and respiration rate, were normal. Her past medical and psychosocial history was unremarkable, as well as her family health history. Brain magnetic resonance imaging (MRI) performed 4 days after clinical onset showed a right frontal-parietal hemorrhagic lesion (Figure 1A). After 10 days, she underwent surgery and gained clinical improvement; however, an inconclusive histological diagnosis was obtained due to the presence of hemorrhage and fibrin clots.

After 3 months (Time 3), the patient reported headache and aggressive mood that lasted for a few weeks; three new brain lesions (bilateral frontal and right parietal-occipital) were identified on MRI. The left basal frontal nodule (Figure 1A) was excised, and the headache resolved. Pathological examination revealed a proliferating vascular lesion with vascular channels, smooth muscle proliferation into the lumen of vessels, and clusters of papillary endothelial hyperplasia positive for CD31 and CD34 endothelial markers (out of the remaining antibodies CD68, CK (AE1/AE3), MIB-1, and Factor VIII). The patient was diagnosed with hemangioendothelioma (HE).

After 4 months of the initial visit (Time 4), the patient presented with acute-onset right arm palsy; a brain MRI revealed a new lesion in the left motor cortex (Figure 1A). She promptly underwent surgery, and the new lesion was resected. Histological examination was performed using the same approach as before, and it revealed wide-ranging hemorrhage and a fibrous tumor wall with fibroblasts lined by flattened cells positive for CD31 and CD34. There was also a central proliferation of endothelial cells with papillary architecture. These features were also interpreted as HE according to the previous diagnosis.

After 1 month (Time 5), considering the number of lesions and their evolution, and based on anecdotal cases, treatment with IFN-α (3 million UI × 3/week) was started and continued for 12 months. She showed good tolerability to the treatment and experienced grade 2 leukopenia only and no further complications.

The patient was radiologically and clinically stable until the 20th month from the beginning (Time 20) when she presented with acute severe dizziness. MRI revealed a right hemispheric cerebellar mass with radiological features similar to that of the previous lesions (Figure 1A). Due to the mass effect of the symptomatic clue, we opted for exeresis, followed by re-challenge therapy with IFN-α for 2 years, from the 21st to the 39th month (Time 21–39). The disease was stable during treatment, then IFN-α was stopped, and no changes occurred in the 3 following years. Histopathological analysis revealed a partially organized hematoma.

After 80 months of the initial onset (Time 80), the patient presented with acute severe headache, confusion, and memory impairment. Brain MRI revealed a large hemorrhagic left frontal lesion (Figure 1A). The patient underwent a surgical procedure, and clinical benefit was obtained. Histological analysis showed a network of papillary structures with an inner dense collagenous shaft lined by a single layer of endothelial cells, finally leading to a diagnosis of IPEH.

Histopathological examination of the surgical specimens was performed using hematoxylin*/*eosin and Masson's trichrome staining and immunohistochemical analysis using the abovementioned antibodies.

In addition, previous histological specimens (obtained at time points 3 and 4) were reviewed; nevertheless, due to the hemorrhagic components, a differential diagnosis between HE and IPEH could not be made using these samples.

In two brain MRIs, obtained at the 87th month (Time 87) and 89th month (Time 89) from the onset, new hemosiderin alterations in the right internal subcapsular (Figure 1B, left) and frontal and parietal white matter (Figure 1B, right) showed that three further lesions had occurred, without clinical signs. Based on the close multifocal radiological recurrences, sirolimus (oral, 1 mg/day, every day for 28 days) was initiated; since then, the disease is stable. The last follow-up was performed 43 months into the treatment, and at the 132nd month from the onset (Time 132, Figure 1C). Sirolimus therapy is still ongoing, the drug is well tolerated, and the patient has a good performance status (Karnofsky Performance Status: 90).

**Figure 2:**
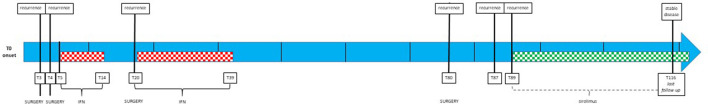
The treatment timeline of the patient. Single-line units in the last 12 months. Dotted squared space: systemic therapy [interferon (IFN), red; sirolimus, green].

Figure 2 illustrates the timeline of the case evolution.

At the onset of the disease and 2 years of follow-ups, an extended check-up was done, including complete blood tests, whole-body computed tomography (CT), scintigraphy, and positron emission tomography, and all results were negative.

## Discussion

IPEH is characterized by endothelial proliferation strictly linked to thrombosis; however, the specific etiopathology of the disease is still largely unknown. It was first described by Masson in 1923 and had been called IPEH since 1976 (3). Recently, excessive intravascular remodeling related to thrombosis or other hemodynamic conditions, resulting in the formation of a papillary pattern, has been proposed as a mechanism of IPEH formation. Based on the lesion location, IPEH is categorized as primary (the most common), which occurs in normal intravascular space; secondary, found within a pre-existing vascular malformation; and extravascular, a third rarer subtype that arises from a hematoma though it has never been reported with intracranial localization (4).

The histological diagnosis of brain-localized IPEH can be challenging mainly due to the destruction of the hemorrhagic component of the lesions, leading to easy misdiagnosis as other conditions. Furthermore, the differential diagnosis includes similar or partially overlapping lesions such as HE, a rare low-proliferation vascular tumor with intermediate behavior between a hemangioma and an angiosarcoma, with possible local aggressivity, occurring in many organs and soft tissues.

There are no specific radiological features that characterize IPEH, and the final diagnosis requires careful histological examination. All tumors appear as mildly hypointense to isointense on T1-weighted images, except for those with recent bleeding which are hyperintense; enhancement on T1 contrasted images is mostly observed; lesions with previous or subacute bleedings show a signal loss on T2^*^ images due to hemosiderin. However, some characteristics of other vascular lesions sustained the hierarchy of differential diagnoses. Hemangiomas can be multiple synchronous or asynchronous, can be small, and show hypointensity in T2^*^ w.i. in addition to hemorrhagic events; they often show caput medusae on enhanced T1 w.i. and calcifications on CT (both features absent in our patient); moreover, such lesions very seldom keep on appearing in a short time in older adults. Cerebral angiosarcoma often shows well-demarcated borders, high-flow serpentine vessels (low signal intensity on T2 weighted images, hyperintensity in T1 and T2 w.i. when thrombotic) in an otherwise solid non-specific soft-tissue mass, cystic or necrotic areas within the lesion (absent in any of our case report lesions), sometimes intralesional calcifications; enhancement can be moderate or strong but mostly heterogeneous and more pronounced on the margin (unlike the lesions in our patient); perilesional edema is often present; angiosarcoma evolves locally with enlarging lesion (contrary to our case) and tends to disseminate. HE is intermediate between angiosarcoma and benign hemangioma because of its tendency to slowly enlarge and metastasize (in fact it was reclassified to grade 3 in the last WHO classification of central nervous system (CNS) tumors while it was considered grade 1 before) within the brain primary hemangioendothelioma and is often a single mass lesion.

Regarding histology, hemangiomas are composed of compact vessels arranged in a multilobular configuration, with inconspicuous to dilated luminal spaces, sclerotic stroma, and vascular walls. HE typically shows well-differentiated nests and cords of cells, often within a myxoid matrix, with abundant eosinophilic cytoplasm and small intracytoplasmic lumina. Angiosarcoma presents irregular vascular channels with poorly differentiated areas, composed of cells characterized by high pleomorphism, atypia, and mitotic activity, and causes more parenchymal destruction than HE while causing less sclerosis. Moreover, HE is characterized by the unique CAMTA1-WWTR1 fusion or YAP1-TFE3 fusion.

Until December 2022, 45 cases of intracranial IPEH have been reported in peer-reviewed literature, mostly as single lesions without parenchymal localization. Out of 45 patients, 18 had secondary cavernous malformation or aneurysm/angioma, often following radiotherapy (5–9). The median age is 37.5 years, ranging from 2 days to 79 years, with a preponderance in adult age (*n* = 37/45). The prevalence is higher in female patients (F:M = 36:9). All the reports are summarized in Table 2. Typically, they are located in the skull base (n = 18/45) or hemispheric (*n* = 18/45: seven frontal, five parietal, two temporal, and four in more than one lobe) and to less extent in the infra-tentorial region (*n* = 4/45: two cerebellar, one brainstem and one posterior inferior cerebellar artery), pineal region (*n* = 2/45), or other location (*n* = 3/45: one para-sellar, one corpus callosum, and one torcular).

**Table 2:** Case reports of intracranial IPEH/Masson's tumor review.

**Case No**.	**References number**	**Age**	**Sex**	**IPEH subtype**	**Lesion location**	**Hem**	**Presenting symptoms**	**Treatment**
1	(4)	56 yrs	F	Primary	Temporal lobe	Yes	Headache, aphasia	GTR
2	(5)	59 yrs	F	Secondary	Skull (cavernosus sinus)	No	Vertigo, gait instability	Partial resection
3	(6)	56 yrs	F	Secondary (AVM)	Parietal lobe	No	Seizures	N.S.
4	(6)	53 yrs	F	Secondary (AVM)	Cingulate	No	Seizures	N.S.
5	(6)	28 yrs	M	Secondary (AVM)	Frontal lobe	No	Headache, focal neurological deficit	N.S.
6	(6)	42 yrs	F	Secondary (AVM)	Parietal lobe	No	None	N.S.
7	(7)	59 yrs	F	Secondary	Temporal	Yes	Seizures, left palsy, headache	GTR
8	(8)	70 yrs	F	Primary	Skull base (cav sinus)	No	Diplopia	STR, RT
9	(8)	51 yrs	F	Secondary (cav mal)	Skull base (cav sinus)	No	CN VI palsy	STR
10	(8)	24 yrs	F	Secondary (cav mal)	Skull base (cav sinus)	No	Diplopia, pituitary dysfunction	GTR
11	(9)	56 yrs	F	Primary	Skull base	No	Chronic sinusitis	STR, RT
12	(9)	39 yrs	F	Primary	Skull base	No	Syncope	GTR
13	(9)	73 yrs	M	Secondary (AVM)	Frontal lobe	No	Seizures	GTR
14	(10)	79 yrs	F	Primary	Pineal	No	Headache	GTR
15	(11)	32 yrs	F	Primary	Frontal lobe	Yes	Aphasia, seizures	GTR
16	(12)	56 yrs	M	Primary	Cerebellar	Yes	Headache, instability, dizziness	GTR
17	(13)	79 yrs	F	Primary	Callosal and intraventricular	Yes	Confusion, instability, incontinence	GTR
18	(14)	51 yrs	F	Primary	Multifocal (frontal, parietal, & occipital lobes)	Yes	Headache, visual field deficit	GTR (occipital lesions only)
19	(15)	11 yrs	M	Primary	Parasellar	No	Visual deficit	STR, RT, chemo (interferon)
20	(16)	12 days	F	Primary	Skull base (mid fossa)	Yes	Hydrocephalus, increased ICP, proptosis	STR, chemo (adoxorubicin/dacarabzine)
21	(17)	16 yrs	F	Primary	Frontoparietal lobes	No	Seizures	STR
22	(18)	3.5 mos	F	Secondary	Frontal lobe	No	Increased ICP, seizures	STR
23	(19)	55 yrs	F	Secondary	Parietooccipital lobes	No	Visual field deficit, hemiparesis, seizures	GTR
24	(20)	15 yrs	F	Primary	Torcula	No	Headache, increased ICP	GTR
25	(21)	27 yrs	F	Secondary (venous angioma)	Skull base (superior orbital fissure)	No	Headache, CN III, V, & VI palsies	GTR
26	(22)	18 yrs	F	Secondary (AVM)	Frontal lobe	Yes	Hemiparesis, seizures	GTR
27	(23)	27 yrs	M	Primary	Skull base	No	Facial weakness	GTR
28	(24)	75 yrs	F	Primary	Skull base	No	Vertigo, hearing loss	STR, Embo, RT
29	(25)	29 yrs	F	Primary	Brainstem	No	Headache, gait imbalance	GTR
30	(26)	46 yrs	F	Secondary (PICA aneurysm)	PICA	No	Otalgia	Aneurysm clipping, GTR
31	(27)	54 yrs	F	Primary	Skull base	Yes	Headache, aphasia	STR
32	(28)	6 yrs	F	Secondary (cortical dysplasia)	Frontoparietal lobes	No	Scalp swelling, seizures	GTR
33	(29)	16 yrs	F	Secondary	Skull base (cav sinus)	No	CN III & VI palsies	STR
34	(29)	18 yrs	F	Primary	Skull base (cav sinus)	No	CN VI palsy	STR
35	(29)	24 yrs	F	Primary	Parietal lobe	No	Headache, seizures	GTR
36	(29)	28 yrs	F	Primary	Skull base (cav sinus)	No	CN III, V1, V2, & VI palsies	GTR
37	(30)	49 yrs	F	Primary	Skull base (petrous bone & jugular foramen)	No	Hearing loss, facial weakness	Embo, STR
38	(31)	41 yrs	F	Primary	Skull base (cav sinus)	No	CN V & VI palsies	STR, RT
39	(32)	50 yrs	F	Secondary (AVM)	Parietal lobe	Yes	SAH, headache	GTR
40	(33)	2 days	M	Primary	Skull base	Yes	Increased ICP, proptosis	Biopsy
41	(34)	72 yrs	F	Secondary	Cerebellar	No	Cerebellar syndrome	GTR
42	(35)	3 mos	M	Primary	Parietal lobe	No	Scalp swelling	GTR
43	(36)	22 yrs	M	Primary	Pineal gland	No	Headache, inattention	STR
44	(37)	24 yrs	M	Primary	Frontal lobe	No	Headache, dizziness	GTR
45	(38)	51 yrs	F	Primary	Skull base	No	Headache, paresthesia, diplopia	Embo, STR

AVM, Arterial Vascular Malformation; chemo, Chemotherapy; cav sinus, cavernous sinus; CN, Cranial Nerve; Embo, Embolization; GTR, Gross Total Resection; ICP, Intra Cranial pressure; mos, months; N.S., Not Specified; PICA, posterior inferior cerebellar artery; RT, Radiotherapy; SAH, Subarachnoid hemorrhage; STR, Sub Total Resection.

Based on the monoistitutional database, Roach et al. (6) estimated an incidence of ~3.9% of IPEH after stereotactic radiosurgery for arterial vascular malformation, occurring after a latent period of up to 10 years. The suspicion of IPEH transformation was generally formulated based on the change in the seizure frequency or a new neurologic deficit accompanied by characteristic radiologic features.

Most patients underwent surgery as initial treatment, and in a few cases, radiotherapy was performed on recurrence. Unfortunately, most reported cases had a short follow-up (13).

We describe a CNS parenchymal primary IPEH presenting with focal neurological signs, which is reported in <40% of cases with parenchymal brain IPEH. To the best of our knowledge, only one multifocal parenchymal case has been reported. In that case, a woman experienced headache and focal signs and underwent exeresis of one lesion, with no progression of disease at the 6-month follow-up (14). Others reported multifocal IPEH that localized outside the CNS (39).

Treatment data displayed in the literature are limited to case report/series, most of which describe single-lesion cases or have a very short follow-up. The treatment of choice for IPEH is surgery, eventually associated with embolization and/or radiotherapy. In two pediatric cases only, systemic therapy has also been reported: Sim et al. described the case of a single para-sellar IPEH treated twice by surgery followed by adjuvant IFN-α, while Sickler et al. reported a case of middle cranial fossa probable IPEH treated with surgery and doxorubicin plus dacarbazine (15, 16).

Our case is noteworthy because of the consecutive multifocal incurrence of exclusively parenchymal brain lesions with a long follow-up (132 months). The approach used to study and the follow-up of the patient were comprehensive, including brain imaging (CT and MRI) as well as total body investigation. We did not proceed with cerebral angiography due to the decision to perform surgical excision of the hemorrhagic symptomatic lesions.

We needed to attempt a systemic treatment strategy for the patient to avoid the collateral effects of consecutive repetitive surgeries and eventual large-dose radiotherapy.

Due to the initial diagnosis of HE, we opted for IFN-α treatment, according to the literature (40). Previous reports suggested IFN-α therapy due to the anti-angiogenic properties of the drug and its analogous indication for similar diseases, including aggressive angiosarcoma (41). Alternative options include cytotoxic chemotherapy, mTOR inhibitors, and bevacizumab-based therapy (42).

The disease evolution in our patient subsequently led to the diagnosis of IPEH. Based on this, combined with the recurrent multifocal brain evolution and the good performance, the treatment decision shifted to sirolimus, an old drug repurposed for anti-proliferative action, already experimented for “extensive and/or complex slow-flow vascular malformations” (NCT01811667) (43). Sirolimus, also known as rapamycin, was initially approved by the US Food and Drug Administration to treat lymphangioleiomyomatosis and for liver and kidney transplants as an immunosuppressive drug, while its role as an anti-cancer and anti-angiogenic agent was later determined. Rapamycin is a specific inhibitor of mTOR, a molecule that controls several processes involved in cell growth and proliferation. These include protein, lipid, and nucleotide synthesis, and the resultance is a suppressive effect on the proliferation, invasion, and metastasizing of tumor cells. The anti-angiogenic properties are mostly related to the suppression of vascular endothelial growth factor signal transduction. In this setting, sirolimus provides anti-cancer benefits in different types of cancer, vascular malformations, and vascular tumors (including aggressive or borderline vascular tumors), with an acceptable safety profile over a wide dose range (44, 45). The most frequently reported side effects are asthenia, oral mucositis, dyslipidemia, leukopenia, gastrointestinal symptoms, cutaneous alterations, and infectious complications. The use of a low dose of sirolimus (1–4 mg daily) has been previously proposed based on the observation that the drug was still effective in patients with vascular anomalies, with a higher safety profile (46). As reported in the literature, time to response may vary among patients although the maximal effect does not occur in a short time; thus, the treatment duration is not defined and must be determined on a case-by-case basis (46).

The case we described got recurrent surgical exeresis and corresponding histology, associated with dedicated neuroimaging, thus allowing an extensive study; we then approached it with systemic therapy, resulting in success. However, as in any case report, this is not enough to prove the efficacy of IFN-α and sirolimus in such a context, and the data need to be replicated.

## Conclusion

In the case described herein, sirolimus, which was started after close multifocal recurrences of brain IPEH, enabled disease stabilization over 43 months of ongoing treatment and for 132 months after onset (43, 44).

The patient has tolerated the long-term low-dose sirolimus treatment well.

The challenging diagnostic and therapeutic approach reported is anecdotic, but it may be helpful in patients affected by such rare pathological entities.

## Data availability statement

The raw data supporting the conclusions of this article will be made available by the authors, without undue reservation.

## Ethics statement

Ethical review and approval was not required for the study on human participants in accordance with the local legislation and institutional requirements. Written informed consent for participation was not required for this study in accordance with the national legislation and the institutional requirements. Written informed consent was obtained from the individual(s) for the publication of any potentially identifiable images or data included in this article.

## Author contributions

EA: drafting/revision of the manuscript for content, including medical writing for content, study concept or design, and analysis or interpretation of data. BP and MS: analysis or interpretation of data. PF, DA, GD, and FF: drafting/revision of the manuscript for content and including medical writing for content. LC: study concept or design. VC: drafting/revision of the manuscript for content, including medical writing for content, major role in the acquisition of data, and analysis or interpretation of data. All authors contributed to the article and approved the submitted version.
